# Prevalence of hepatitis B surface antigen (HBsAg) positivity and its associated factors in Rwanda

**DOI:** 10.1186/s12879-019-4013-4

**Published:** 2019-05-03

**Authors:** Jean Damascene Makuza, Jean Olivier Twahirwa Rwema, Corneille Killy Ntihabose, Donatha Dushimiyimana, Justine Umutesi, Marie Paul Nisingizwe, Janvier Serumondo, Muhamed Semakula, David J. Riedel, Sabin Nsanzimana

**Affiliations:** 1 IHDPC Department, Rwanda Biomedical Center, Po Box 7162, Kigali, Rwanda; 2 Department of Epidemiology, Johns Hopkins Bloomberg School of Public Health, Baltimore, USA; 3 Institute of Human Virology, University of Maryland School of Medicine, Baltimore, USA; 4 School of Population and Public Health, University of British Columbia, Vancouver, Canada

**Keywords:** Hepatitis B, Prevalence, Risk factors, Rwanda

## Abstract

**Background:**

The epidemiology of hepatitis B virus (HBV) infection in the general population in Rwanda is not well known. This study examined the prevalence of HBV surface antigen (HBsAg) positivity and associated risk factors among people aged 25 years and over in an organized national screening campaign.

**Methods:**

This is a cross-sectional study using data from a nationwide HBV screening campaign organized by the Rwanda Biomedical Centre from March to October 2018. This campaign targeted individuals aged > 25 years old from 24 of 30 districts of Rwanda. Sensitization was done through multimedia announcements, community health workers and local church leaders. During the campaign, a structured interview was administered by trained healthcare workers to collect information on socio-demographic, clinical and behavioral characteristics of participants; HBV screening was performed with HBsAg using enzyme-linked immunosorbent assays (ELISA) testing. Bivariate and multivariate logistic regressions were used to assess factors associated with HBsAg positivity in the screened participants.

**Results:**

A total of 327,360 individuals were screened during the campaign. Overall 12,865(3.9%) were HBsAg positive. The highest prevalence (4.2%) was found in the 35–44-year-old group, but the difference from other groups was not significant (Odds Ratio [OR = 1.057, 95% Confidence Interval(CI) (0.904–1.235)]. Being male [OR = 1.348, 95% CI (1.30,1.40)]; being single [OR = 1.092, 95% CI (1.10–1.16)] compared to married; a previous positive TB screening test [OR = 2.352, 95% CI (1.63–3.39)]; history of surgical operation [OR = 1.082, 95% CI (1.00,1.17)]; exposure to traditional operational practices and scarification [OR = 1.187, 95% CI (1.13, 1.24)]; and having a person in the family with viral hepatitis [OR = 1.367, 95% CI (1.21, 1.53)] were significantly associated with HBV infection.

**Conclusions:**

These data provide the first national estimate of the prevalence of HBsAg seropositivity and its associated factors in Rwanda. The study identified people with the highest risk of HBV infection who should be the priority of future prevention efforts in Rwanda and in similar settings.

**Electronic supplementary material:**

The online version of this article (10.1186/s12879-019-4013-4) contains supplementary material, which is available to authorized users.

## Background

Viral hepatitis caused an estimated 1.34 million deaths in 2015 worldwide [1]. These deaths were as many as those caused by tuberculosis and higher than those caused by HIV [2]. Most (720,000) hepatitis related deaths were due to liver cirrhosis, followed by primary liver cancer i.e. hepatocellular carcinoma (470,000 deaths) [3]. Globally, an estimated 257 million people were living with chronic hepatitis B infection (HBV) in 2015, and the global prevalence of HBV infection in the general population was 3.5% [4]. However, the prevalence of chronic HBV infection is much higher among people born before the HBV vaccine became available [5].

Despite the introduction of universal HBV vaccination and effective antiviral therapy, the estimated overall seroprevalence of HBV surface antigen remains high in Africa at 6.1% [6] and the Western Pacific regions (6.2%) [1]. According to the World Health Organization (WHO), HBV infection affects more than 5% of the local population in sub-Saharan Africa, with more than 8% in West Africa and reaching up to 15% in some areas [7]. In East African countries, only a few studies have been conducted on the epidemiology of viral hepatitis, and most of these studies focused on specific subpopulations, e.g. those living with HIV [8–13]. Data for the general populations are mostly unavailable.

In Rwanda, HBV prevalence data is only available among specific population subgroups. One study among 13,121 pregnant women in 30 sentinel sites found a prevalence of HBsAg of 3.7% [8]. A recent study of 117,258 people living with HIV (PLHIV) found a prevalence of HBsAg of 4.3% [10]. However, there are limited data on the epidemiology of HBV in the general population.

In order to better understand the seroprevalence of HBV in the general population of the country, the Rwanda Biomedical Center (RBC) and its partners conducted several HBV screening campaigns to inform HBV prevention and treatment programming in Rwanda. This article analyzed the prevalence and risk factors of HBsAg seropositivity among people who participated in a national hepatitis screening campaign in Rwanda.

## Methods

### Study design

This is a cross-sectional study. Data collection was done at the time of HBV screening using a standardized data collection tool administered by trained health care providers.

### Study population and recruitment of participants

This campaign was conducted from 29 March to 26 October 2018 in 24 districts in Rwanda. These districts had been identified as having a high prevalence of HBV in an earlier brief campaign in 2017 that was conducted in all the 30 districts. The study population consisted of individuals aged 25 years and above. Sensitization on HBV screening and participants’ mobilization was done through radio advertisements and with the help of community health workers before and during the screening period. All participants who attended screening sites (health centers) were screened for HBV after verbal consent was obtained.

### Data collection procedures

A chart abstraction instrument (Additional file 1) was elaborated for collecting socio-demographic, clinical and behavioral data. Trained healthcare providers used registers of participants to complement information gathered from the interviews. Blood sample collection for HBV screening was performed by the same trained nurses and laboratory technicians. Data were extracted from laboratory request forms stored in the testing sites and entered into a database by trained health care providers.

HBV screening was done by laboratory technicians with experience in enzyme-linked immunosorbent assays (ELISA) testing. Prior to the screening campaign, a 2-week practical training session was organized for the laboratory technicians. The following procedures were followed for screening: specimens were collected at the site of enrollment and then transported to testing facilities. Testing was performed at 13 laboratories which were all 5-star accredited satellite laboratories serving as hubs of the National Reference Laboratory and distributed across all provinces of Rwanda. The 5-star laboratories are ones that achieved 95% of WHO evaluation for accreditation to provide an interim pathway for measuring, monitoring, and recognizing improvement toward the realization of international laboratory standards and subsequent application to full International Organization for Standardization (ISO) 15,189 schemes [14]. Tests were conducted using Murex ELISA for HBsAg (version 3.0). No confirmatory tests were done. All testing procedures were supervised by a team of laboratory technicians from the National Reference Laboratory of Rwanda. Data entry was done using Microsoft Excel 2013.

### Variables

The primary outcome was a binary variable of HBsAg positivity, defined as “positive” if participants tested HBsAg positive, and “negative” if participants tested HBsAg negative. Independent variables were socio-demographic characteristics including age, sex, screening district, type of health insurance, and socioeconomic status (Ubudehe category). Ubudehe is a home-grown development program whereby citizens are placed into different categories by socioeconomic status as defined in accordance with Ministry of Local Government (MINALOC) criteria: communities periodically rank households on a scale of 1 to 4 according to their perceived poverty and vulnerability status, with a score of 1 being the most vulnerable and 4 the least vulnerable [15].

Clinical and behavioral variables were also assessed including high blood pressure, diabetes, chronic renal failure, cancer, tuberculosis, surgical operation, unhygienic traditional surgical operation practices (these include scarification, uvulectomy, intentional cutting of the body known as indasago, tattoos, and traditional dental extraction), history of blood transfusion, multiple sex partners, history of viral hepatitis in the family, and HIV status. Variables were all self-reported by participants.

### Statistical methods & data analysis

After data cleaning using Excel, they were imported and analyzed using Statistical Package for the Social Sciences (SPSS) version 20.0. In the bivariate analysis, logistic regression was used to assess the association between categorical variables and outcome. Variables that were statistically significant at the 5% level of significance were retained in the final model building. In the multivariate analysis, potential determinants of HBsAg positivity were assessed using logistic regression. Variables that were not significant were eliminated using backward stepwise method. The study considered 326,100 of 327,360 participants screened in the logistic regression analysis; 1260 participants were removed due to missing values.

### Ethics

Routinely collected program data analyzed for this study are maintained by the Rwanda Biomedical Centre (RBC), Division of HIV/AIDS, STIs and Other Blood Borne Infections. The ethical procedures for the collection of these data were governed by the Medical Research Council of Rwanda, and site authorizations were obtained from the Ministry of Health for hosting sites. Secondary analyses of routinely collected data are exempt by RBC. Approval (No. 2048/RBC/2019) for utilization of the data was obtained by RBC. Verbal consent was obtained from all eligible participants to HBV screening before blood sample and interview based data collection and ethical review approval was conferred due to the routine nature of the data.

## Results

### Study population

**Fig. 1:**
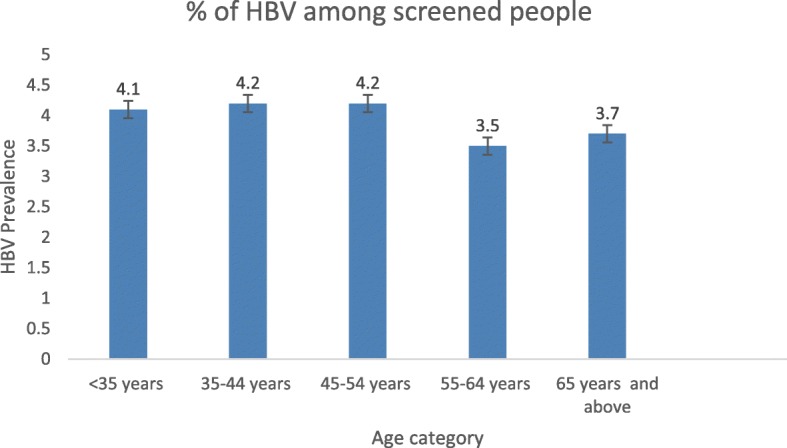
Prevalence of HBs Ag by age category of Participants

Socio-demographic characteristics of this study population are shown in Table 1. The mean age of participants was 44.8 years [44.74–44.84] with the majority of participants 87,554(25%) in the 25–34-year-old group as shown in Fig. 1. The majority (68.8%) were female, and 77.5% of participants were married. Nearly half (45.5%) of participants were in Ubudehe category 3, and nearly all (93.6%) participants were enrolled in the community-based health insurance (mutuelle). Among all participants, 5.6% had been operated on at least once and 16.6% had a history of traditional surgical and scarification practice.

**Table 1:** General characteristics of participants in a national HBV screening campaign in Rwanda

Characteristics	All participants N (%)	HBsAg positive N (%)	HBsAg negative N (%)
Gender(*N* = 326175)			
Female	224,382(68.8)	7996(3.6)	216,386(96.4)
Male	101,793(31.2)	4813(4.3)	96,980(95.7)
Age group (*N* = 319302)			
< 35 years old	91,922(28.8)	3737(4.1)	88,185(95.9)
35–44 years old	79,337(21.8)	3321(4.2)	76,016(95.8)
45–54 years old	61,733(21.5)	3321(4.2)	58,412(95.8)
55–64 years old	50,379(18.3)	1782(3.5)	48,597(96.5)
65 years old and above	32,965(11.6)	1229(3.7)	31,736(96.3)
Marital status (N = 319,302)			
Married	247,437(77.5)	1217(4.3)	246,220(95.7)
Single	28,188(8.8)	9813(4.0)	18,375(96.0)
Widow	33,994(10.6)	1213(3.6)	32,781(96.4)
Divorced	1644(0.5)	53(3.2)	1591(96.8)
Separated	8039(2.5)	298(3.7)	7741(96.3)
Ubudehe category (*N* = 318855)			
Category 1 (most vulnerable)	49,886(15.6)	1849(3.7)	48,037(96.3)
Category 2	119,643(36.5)	4706(3.9)	114,937(96.1)
Category 3	149,092(45.5)	5950(4.0)	143,142(96.4)
Category 4 (least vulnerable)	234(0.1)	12(5.1)	222(94.9)
Health Insurance*(*N* = 327360)			
Mutuelle	303,206(93.6)	11,927(3.9)	291,279(96.1)
RAMA	14,708(4.5)	569(3.9)	14,139(96.1)
MMI	2574(0.8)	109(4.2)	2465(95.8)
MEDIPLAN and other private insurances	3465(1.1)	132(3.8)	3333(96.2)
Province of origin((*N* = 315040)			
South	48,994(15.6)	2471(3.8)	46,523(96.2)
West	89,885(28.5)	3415(3.8)	86,470(96.2)
North	65,824(20.9)	1648(3.4)	64,176(96.6)
East	110,337(35.0)	4783(4.3)	105,554(95.7)
Diabetes (*N* = 326926)			
No	325,059(99.4)	12,765(3.9)	312,294(96.1)
Yes	1867(0.6)	82(4.4)	1785(95.6)
Hypertension (*N* = 326931)			
No	317,421(97.1)	12,480(3.9)	304,941(96.1)
Yes	9510(2.9)	370(3.9)	9140(96.1)
Chronic Renal Failure (*N* = 326930)			
No	326,323(99.8)	12,648(3.9)	313,675(96.1)
Yes	578(0.2)	202(4.9)	376(95.1)
Cancer (*N* = 326901)			
No	32,016(98.0)	12,821(3.9)	19,195(96.1)
Yes	293(0.2)	27(4.7)	266(95.3)
HCV infection (*N* = 326193)			
Negative	313,355(96.1)	12,061(4.0)	301,294(96.0)
Positive	12,838(3.9)	777(3.5)	12,061(96.5)
HIV status (*N* = 326913)			
Negative	320,316(98.0)	12,570(3.9)	307,746(96.1)
Yes	6597(2.0)	279(4.2)	6318(95.8)
Ever had TB (*N* = 326919)			
No	326,581(99.9)	12,813(3.9)	313,768(96.1)
Yes	338(0.1)	35(10.4)	303(89.6)
Ever been operated (*N* = 326913)			
No	309,037(94.4)	12,066(3.9)	296,971(96.1)
Yes	17,876(5.6)	781(4.4)	17,095(95.6)
Ever been transfused (*N* = 326922)			
No	321,572(98.4)	12,590(3.9)	308,982(96.1)
Yes	5350(1.6)	258(4.8)	5092(95.2)
Traditional operation and scarification (*N* = 326652)			
No	272,555(83.4)	10,363(3.8)	262,192(96.2)
Yes	54,097(16.6)	2477(4.6)	51,620(95.4)
Having more than 1 sexual partner (*N* = 326785)			
No	318,030(97.3)	12,428(3.9)	305,602(96.1)
Yes	8755(2.7)	416(4.8)	8339(95.2)
Viral Hepatitis in the family (*N* = 326882)			
No	320,692(98.1)	12,494(3.9)	308,198(96.1)
Yes	6190(1.9)	353(5.7)	5837(94.3)

* Mutuelle: Community based health insurance

*RAMA: La Rwandaise Assurance Maladie: Health insurances for employees of public and private sectors

*MMI: Military Medical Insurance

*MEDIPLAN: Private insurance initiated by Insurance Company called SORAS

Table 2 shows the prevalence of HBsAg according to different characteristics among participants. The overall prevalence of HBsAg was 3.9% (12,865/326,652). HBsAg positivity was most prevalent in the 35–44 year-old and 45–54-year-old age groups (4.2% in both; see also Fig. 1), in males (4.3%), in participants in Ubudehe class 4 (5.1%).

**Table 2:** Prevalence of HBsAg according to different characteristics among participants in 24 districts in Rwanda

Characteristics	Frequencies		Bivariate analysis		Multivariate analysis	
	Frequency	HBsAg positive (%)	OR	(95% CI)	OR	(95% CI)
Overall HBV prevalence (*N* = 327360)						
Negative	314,420	96.1				
Positive	12,865	3.9		(3.83–3.97)		
Gender (*N* = 326100)						
Female	224,335	7996(3.6)	1		1	
Male	101,765	4813(4.3)	1.343	(1.295–1.393)	1.353	(1.301–1.408)
Age group (*N* = 317356)						
< 35 years	92,220	3737(4.1)	1		1	
35–44 years	79,638	3321(4.2)	1.030	(0.982–1.081)	1.039	(0.988–1.105)
45–54 years	61,889	3321(4.2)	0.956	(0.908–1.008)	0.976	(0.923–1.109)
55–64 years	50,544	1782(3.5)	0.865	(0.817–0.917)	0.895	(0.841–0.952)
65+ years	33,065	1229(3.7)	0.914	(0.856–0.976)	0.923	(0.861–0.965)
Marital status (*N* = 319229)						
Single	28,184	1217(4.3)	1		1	
Married	247,377	9813(4.0)	0.915	(0.861–0.973)	1.064	(0.975–1.161)
Widow, Divorced and separated	43,668	15,643.6)	0.823	(0.763–0.889)	0.985	(0.927–1.047)
Ubudehe category (*N* = 318784)						
Category 1	49,881	1849(3.7)	1		1	
Category 2	119,622	4706(3.9)	1.051	(1.007–1.124)	1.019	(0.994–1.112)
Category 3	149,047	5950(4.0)	1.053	(1.024–1.139)	1.03	(0.996–1.114)
Category 4	234	12(5.1)	1.372	(0.784–2.515)	2.127	(0.765–2.463)
Health Insurance(*N* = 323879)						
Mutuelle	303,140	11,927(3.9)	1		1	
RAMA	14,701	569(3.9)	0.983	(0.902–1.071)	0.893	(0.815–0.978)
MMI	2573	109(4.2)	1.08	(0.891–1.310)	1.013	(0.828–1.238)
MEDIPLAN and other private insurances	3465	132(3.8)	0.967	(0.812–1.152)	0.874	(0.717–1.067)
Province of screening (*N* = 313945)						
North	65,812	2471(3.8)	1		1	
West	89,857	3415(3.8)	1.013	(0.961–1.068)	0.993	(0.941–1.049)
South	48,963	1648(3.4)	0.893	(0.863–0.951)	0.956	(0.892–1.025)
East	110,334	4783(4.3)	1.162	(1.105–1.221)	1.132	(1.075–1.192)
Diabetes (*N* = 326,851)						
No	324,985	12,765(3.9)	1.000			
Yes	1866	82(4.4)	1.124	(0.900–1.404)		
High Blood pressure (*N* = 326856)						
No	317,348	12,480(3.9)	1.000			
Yes	9508	370(3.9)	0.989	(0.890–1.099)		
CRF (*N* = 326855)						
No	322,718	12,648(3.9)	1.000			
Yes	4137	202(4.9)	1.258	(1.091–1.451)		
Suffering from Cancer (*N* = 326826)						
No	326,248	12,821(3.9)	1			
Yes	578	27(4.7)	1.198	(0.814–1.764)		
HCV infection(326193)						
Negative	313,355	12,061(4.0)	1		1	
Positive	12,838	777(3.5)	0.879	(0.816–0.946)	0.880	(0.812–0.924)
HIV status (*N* = 326838)						
Negative	320,242	12,570(3.9)	1		1	
Positive	6596	279(4.2)	1.081	(0.958–1.220)	0.835	(0.541–1.290)
Ever had TBC (*N* = 326844)						
No	326,506	12,813(3.9)	1.000		1.000	
Yes	338	35(10.4)	2.828	(1.992–4.015)	2.352	(1.634–3.385)
Ever been operated (*N* = 326838)						
No	308,966	12,066(3.9)	1.000		1.000	
Yes	17,872	781(4.4)	1.124	(1.044–1.211)	1.082	(1.000–1.171)
Ever been transfused (*N* = 326847)						
No	321,500	12,590(3.9)	1.000		1.000	
Yes	5347	258(4.8)	1.244	(1.096–1.411)	1.122	(0.980–1.284)
Ever been traditionally operated (*N* = 326577)						
No	272,493	10,363(3.8)	1.000		1.000	
Yes	54,084	2477(4.6)	1.214	(1.161–1.270)	1.187	(1.133–1.244)
Having more than 1 sexual partner (*N* = 326710)						
No	317,958	12,428(3.9)	1.000		1.000	
Yes	8752	416(4.8)	1.227	(1.110–1.356)	1.074	(0.968–1.191)
Viral Hepatitis in the family (*N* = 326807)						
No	320,622	12,494(3.9)	1.000		1.000	
Yes	6185	353(5.7)	1.493	(1.339–1.665)	1.367	(1.221–1.531)

In the multivariable analysis, factors that remained statistically significantly associated with HBsAg positivity were male sex OR = 1.34 [95% CI 1.30–1.40]; living in the Eastern province OR = 1.132 [95% CI 1.075–1.192]; being single OR = 1.092 [95% CI 1.10–1.16] compared to married; having a history of tuberculosis OR = 2.352 [95% CI 1.63–3.39], surgical operation OR = 1.082 [95% CI 1.00–1.17], or traditional operational practices and scarification OR = 1.187 [95% CI 1.13–1.24]; and a history of viral hepatitis in the family OR = 1.367 [95% CI 1.21–1.53].

## Discussion

This study was the first national-level study of HBsAg prevalence in Rwanda and showed that the overall prevalence of HBsAg positivity was 3.9% in the screened population. This prevalence is comparable to the 4.3% prevalence among PLHIV and the 3.7% prevalence among pregnant women in Rwanda [8, 10]. From 2015 in Rwanda, there have been several wide scale vaccination campaigns among people at risk for HBV; these campaigns may have lowered the prevalence in adults. These findings indicate that the Rwandan HBV prevalence is intermediate according to WHO criteria, as the prevalence ranges between 2 and 8% [16]. Nearby East African countries also have prevalence ranges estimated at 2–8%. For example, Kenya, Tanzania, and Ethiopia have general population prevalence reported at 2, 6, and 8%, respectively [4, 13, 17].

In addition to estimating the prevalence of HBV infection in the population screened, this study has identified different factors associated with prevalent HBV infection. Identification of risk factors is essential as it informs HBV prevention programming especially in resource-limited countries [18]. The factors associated with HBsAg positivity in this study included male sex, province of origin, and some clinical and behavioral characteristics including history of tuberculosis, exposure to blood transfusion, and viral hepatitis in the family. These factors have also been identified to be associated with HBV infection in other studies. Many of the identified factors have very low odds ratios (and may have been overpowered given the large sample size).

Male sex was one of demographic characteristics associated with HBsAg positivity in this study. The reasons behind this association may be that males may be more prone to high risk behaviors like sexual contact, violence and conflicts in which blood contact may occur. Other studies from Pakistan [19] and Tanzania [13] also found that male sex was associated with HBV infection. In Rwanda, a prior study conducted among PLHIV also found higher HBV infection among men [10].

**Fig. 2:**
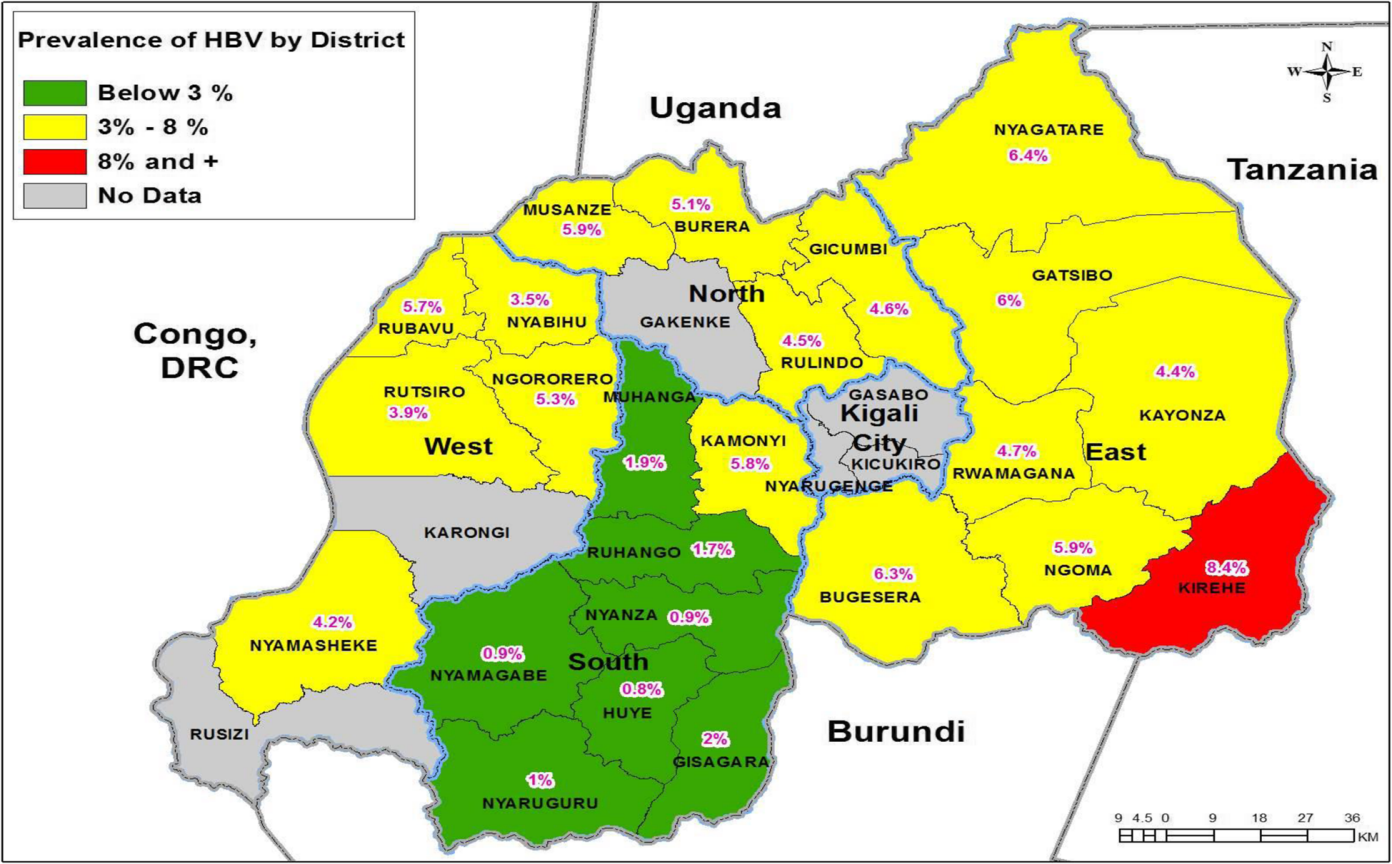
Geographical repartition of prevalence of HBs Ag in Rwanda. B Kurujyishuri , JD Makuza et al. Kigali, Dec 2019.

High HBsAg prevalence was found in Eastern province with the peak prevalence in Kirehe district (8.4%) as presented in Fig. 2. The Eastern province of Rwanda has a large number refugees and other migrants from bordering countries. It is possible that this population has a higher risk of HBV acquisition due to unhygienic living conditions, low vaccination rates, or possibly behaviors (like traditional scarification). The Eastern Province has now been targeted for future HBV vaccination campaigns.

Among risks factors found in this study, certain clinical characteristics like a history of tuberculosis, surgical operation, and traditional operational practices and scarification and a history of viral hepatitis in the family were found to be associated with HBsAg positivity. Being exposed to blood and blood products through medical and non-medical practices is the main source of HBV transmission and could be the reason why these factors were associated with HBV infection in this study. Previous studies have identified exposure to blood products, history of surgical operations, history of HBV in the family, undergoing body piercing as risks factors for HBV [20–25]. In a study conducted in Sao Paulo, Brazil HBV infection was associated with a family history of hepatitis and tattoos practicing [26] were reported as main factors associated with HBV. Over the last decade, Rwanda has made great strides in securing and testing the blood supply, and it is though that iatrogenic HBV exposures should continue to decline.

The results from this study provide an estimate of the prevalence of HBV infection in Rwanda based on a large number of diverse participants from different districts in the country. These estimates help to inform HBV prevention and treatment programming in Rwanda while waiting for more generalizable prevalence estimates from studies conducted on random national samples. Additionally, identification of factors associated with HBV in this population can allow the design and implementation of interventions targeted to the most at risk populations in Rwanda. For instance, organizers of HBV screening, prevention and treatment programs could prioritize groups that were identified to be most at-risk including people with a history of TB, transfusions, and those from families with a history of HBV infection.

This study has strengths and several limitations. The study strength is its very large sample size and high coverage of 24 of the 30 districts in Rwanda (80%). However, the study population was not randomly selected and did not include Kigali city, so the prevalence results may not be generalizable to the whole country. There is also a possibility of selection bias, as people who participated in the screening program may have been more likely to have current symptoms, already know their HBV status, or be more likely to be HBV-infected. This study was aimed at individuals aged 25 years and older; the prevalence of HBV is likely higher in this group than in those under 25 years in Rwanda, as childhood HBV vaccination programs were begun in 2002. Future studies will need to determine the prevalence among the population less than 25 years old. Another bias may have been introduced by encouraging older individuals to come for testing, which may have resulted in an unequal age distribution. This study relied on chart review and self-reported information for clinical variables (e.g. HIV status) which may have led to misclassification through recall bias. There were also a limited number of variables available for analysis in the chart (e.g. HBV vaccination status), limiting the number of factors and confounders that could be included in the analysis. Finally, this study relied on HBsAg testing to determine prevalence of active HBV infection. Therefore, participants with acute infection or other chronic carriers may have been missed, thus underestimating the total burden of HBV disease. Without liver function testing or fibrosis data, subjects could not be assessed for the impact of chronic HBV infection. Despite these limitations, this study provides valuable baseline prevalence estimates to inform HBV programming in Rwanda and identifies groups for focused prevention efforts. Future studies using random sampling techniques may give a more complete estimation of the national prevalence.

## Conclusion

This study shows that HBV is an intermediate epidemic in the studied population in Rwanda. The findings are similar to little available data on HBV in the region and may be applicable in other similar settings. These results have also identified groups of people with the highest HBV risk in Rwanda. More studies are needed in order to have people that should be prioritized in prevention interventions.

## Additional file

Additional file 1: Interview guide. (DOCX 128 kb)

## Abbreviations

CI: Confidence Interval
ELISA: Enzyme-Linked Immunosorbent Assays
GAVI: Global Alliance for Vaccines and Immunization
HBsAg: Hepatitis B Surface Antigen
HBV: Hepatitis B virus
HIV/AIDS: Human Immunodeficiency virus/ Acquired Immunodeficiency Diseases Syndrome
MEDIPLAN: Private insurance initiated by Insurance Company called SORAS
MINALOC: Ministry of Local Government
MMI: Military Medical Insurance
OR: Odd Ratio
PLHIV: People Living with HIV
RAMA: La Rwandaise Assurance Maladie: Health insurances for employees of public and private sectors
RBC: Rwanda Biomedical Center
SPSS: Statistical Package for the Social Sciences
TB: Tuberculosis
WHO: World Health Organization
