# Supplementary material for: Prevalence of hepatitis B surface antigen (HBsAg) positivity and its associated factors in Rwanda
Source: BMC Infect Dis. 2019 May 3;19:381. doi: 10.1186/s12879-019-4013-4 (PMC6499977; doi:10.1186/s12879-019-4013-4)
Supplement: Supplementary file 1 — Interview guide. (DOCX 128 kb) [file 12879_2019_4013_MOESM1_ESM.docx]

| **HBsAg/HCVAb Screening: INTERVIEW GUIDE** | | |
| --- | --- | --- |
| **SECTION 1. To be filled at enrolment facility** | | |
| **Screening Health Facility**: | | **Date of sample**: _____ / _____ / ________ |
| **DEMOGRAPHICS** | | |
| **Patient Name**: | | |
| **Phone number:** | | |
| **District**: | | **Sector**: |
| **Cell**: | | **Village**: |

| **Ubudehe:** | 1 | 2 | 3 | 4 | others |
| --- | --- | --- | --- | --- | --- |

| **Year of Birth**: __________________ | | | | | | | | | | | | | | | |
| --- | --- | --- | --- | --- | --- | --- | --- | --- | --- | --- | --- | --- | --- | --- | --- |
| **Marital Status**: Married single Widow Divorced Separated | | | | | | | | | | | | | | | |
| **Sex**: |  | | | Male | |  | Female | | | | | | | | |
| **Health Insurance**: | | | Mutuelle | | | RAMA | | | | MMI | | | Private | | None |
| **CO-MORBIDITIES** | | | | | | | | | | | | | | | |
| Diabetes: Yes/ No/ Do not know | | | | | | | | | Chronic renal failure: Yes/ No/ Do not know | | | | | | |
| Cancer: Yes/ No/ Do not know | | | | | | | | | HIV Infection: Yes/ No/ Do not know | | | | | | |
| Hypertension: Yes/ No/ Do not know | | | | | | | | | Other, specify: _______ ________________ | | | | | | |
| **Risks of Viral Hepatitis** | | | | | | | | | | | | | | | |
| Ever been operated | | | | | | | | | Ever been traditionally operated (ibyinyo, ibirimi, indasago, scarifications, tattoo, …) | | | | | | |
| Ever transfused | | | | | | | | | Having more than one sexually partner | | | | | | |
| Viral hepatitis in the family | | | | | | | | | Other, specify: _______ ________________ | | | | | | |
| **SECTION 2. To be filled at testing site** | | | | | | | | | | | | | | | |
| **TEST RESULTS SCREENING** | | | | | | | | | | | | | | | |
| **Testing Facility:** | | | | | | | | | | | | **Testing date**: _____ / _____ / ________ | | | |
| **HBsAg result** | | | | Positive | | | | | Negative | | | | | Indeterminate | |
| **Anti-HCV result** | | | | Positive | | | | | Negative | | | | | Indeterminate | |
| **HIV result, if tested** | | | | Positive | | | | | Negative | | | | | Indeterminate | |
| **Feedback** | | | | Refer to vaccination, if HBsAg negative | | | | | | | Refer to care, if HBsAg or anti-HCV positive | | | | |
| **Clinic staff (Enrolment Facility:** ____________________ **Signature:** _______________________ | | | | | | | | | | | | | | | |
| **Lab technician (ELISA Testing):** ____________________ **Signature:** _______________________ | | | | | | | | | | | | | | | |
| **Clinical Mentor (Feedback):** ____________________ **Signature:** _______________________ | | | | | | | | | | | | | | | |
